# Neural processing of laughter in depression

**DOI:** 10.1038/s41598-025-97385-6

**Published:** 2025-04-27

**Authors:** Thomas Ethofer, Silvia Straub, Benjamin Kreifelts, Katharina Koch, Lena Obermeyer, Sophia Stegmaier, Michael Erb, Klaus Scheffler, Dirk Wildgruber

**Affiliations:** 1 University Clinic for Psychiatry and Psychotherapy Tübingen, Calwer Str. 14, 72076 Tubingen, Germany; 2 German Center for Mental Health, Calwer Str. 14, 72076 Tubingen, Germany; 3 Department of Biomedical Magnetic Resonance, Otfried-Müller-Str. 51, 72076 Tubingen, Germany; 4 Department for High-field Magnetic Resonance, Max-Planck-Institute for Biological Cybernetics, Max-Planck Ring 11, 72076 Tubingen, Germany

**Keywords:** Laughter, Social intent, Rating, Depression, fMRI, Anteromedial prefrontal cortex, Human behaviour, Auditory system, Social behaviour, Social neuroscience, Visual system

## Abstract

Laughter can convey social intent ranging from acceptance (friendly inclusive laughter) to rejection (malign taunting laughter). We investigated perception of auditory and visual laughter in patients with major depressive disorder (MDD) versus healthy controls (HC). 48 MDD patients and 52 HC rated 60 laughter recordings presented auditorily or visually regarding the expressed social intent during an fMRI experiment at 3T. Depression severity was assessed based on questionnaires. MDD patients rated the perceived social intent of the laughter significantly more negative than HC across both modalities. The individual magnitude of this negativity bias of social intent attribution significantly correlated with both depression severity as well as activation in anteromedial prefrontal cortex (AMPFC) during perception of auditory laughter. MDD patients also exhibited a significantly reduced activation in AMPFC and depression severity partially mediated effects on rating of auditory laughter as evidenced by mediation analysis. Our results demonstrate altered perception of social intent expressed by laughter in MDD. Neuroimaging data point to the AMPFC for mediation of this effect as its activity was correlated with both depression severity and a negative attribution bias during perception of auditory laughter. Furthermore, at group level activity in this area was reduced in MDD patients.

**Supplementary Information:**

The online version contains supplementary material available at 10.1038/s41598-025-97385-6.

## Introduction

Laughter is an ancient phenomenon which is not restricted to humans^1^. It occurs 30 times more frequent in social than in solitary situations^2^ and this effect can also be observed in chimpanzees which likewise laugh more often during social than solitary play^3,4^. Therefore, laughter represents a social signal and it has been even proposed that “the essential stimulus for laughter is not a joke, but another person”^5^. Laughter fulfils various functions ranging from the reflex-like tickling laughter as an ancient means of communication to more intentional laughter ranging from friendly “laughing with” others intended to promote social coherence to unfriendly or even hostile “laughing at” adversaries^6^.

Early functional magnetic resonance imaging (fMRI) studies on auditory laughter perception indicated preferential responses during perception of laughing versus crying^7,8^ as well as laughter versus speech and nonvocal sounds in auditory areas within the right superior temporal gyrus (STG)^9^. A subsequent study comparing neural responses to different laughter types revealed that the right STG preferentially responds to tickling laughter while social (e.g. joyful or taunting) laughter recruits the anterior medial prefrontal cortex^10^ (AMPFC) and that these two brain regions are associated with connectivity changes depending on laughter type and attention^11^. Similar observations of differential responses of these key areas for laughter perception have been made in further fMRI studies indicating that the AMPFC responds stronger to deliberately emitted laughs than spontaneous laughter while the STG shows the reverse pattern^12^. Activation in the AMPFC additionally showed a correlation with perceived valence^13^ and social intent attribution^14^ which concurs with the pivotal role of this area in social cognition^15^ and emotion regulation^16^.

Social cognition is impaired in major depressive disorder^17^ (MDD) and numerous studies demonstrated this for recognition of facial expressions (for a review, see^18,19^) as well as other socially relevant stimuli including emotional prosody (for a review, see^20^), eye movements (for a review, see^21^), emotional word content^22^, non-verbal affective bursts^23^, and emotional body language^24^.

More specifically, an interpretation bias has been observed in patients with MDD, leading to interpret social cues more negatively^25^. The negativity bias is particularly pronounced for self-referential stimuli, i.e. social signals that are interpreted by the recipient as attributions to the own person^25^ or as an expression of a hostile intention towards the recipient^26^. Negatively biased self-referential processing, in turn, is assumed to reinforce negative self-perception and contribute to the maintenance of depressive symptoms according to cognitive models of the disorder^27,28^.

However, so far, there are no studies available that examine how perception of laughter is changed in MDD and thus the current study aimed to address this aspect of social cognition in MDD. The participants of the current study were asked to rate the social intention (from friendly to hostile) expressed in laughter presented auditorily or visually during fMRI. Behaviorally, the following hypothesis was evaluated: Laughter is perceived as expressing a more negative social intent in MDD patients than healthy participants (between-groups comparison of intent ratings across all stimuli). Moreover, it was analyzed in an exploratory approach, if the magnitude of this negativity bias differs between both modalities (between-groups comparison of behavioral responses for visual and auditory stimuli separately) and if there is a linear relationship between symptoms of depression and ratings of laughter stimuli resulting in more negative responses with increasing depression scores (correlation analysis between behavioral rating and depression severity). At neural level, the main focus of the current study was to examine a possible relationship between brain activation and social intent attribution during perception of auditory and visual laughter. Therefore, we identified areas for evaluation of social intent expressed by laughter using a whole-brain regression analysis between activity and average behavioral rating of social intent across participants. Based on the general role of the AMPFC in social cognition^15^, and its specific role in interpretation of laughter^10,14^ we hypothesized that this area shows a direct relationship between activation and behavior (null hypothesis: the regression slope between AMPFC activation and behavior is not different from zero). In a second step, we examined whether activation in the functionally defined areas differ between MDD patients and healthy controls (HC), whether activation covaries with depression severity (quantified by self-rating questionnaires on depression and anhedonia) and whether activation mediates the relationship between depression and behavior.

## Results

### Behavioral data

**Fig. 1:**
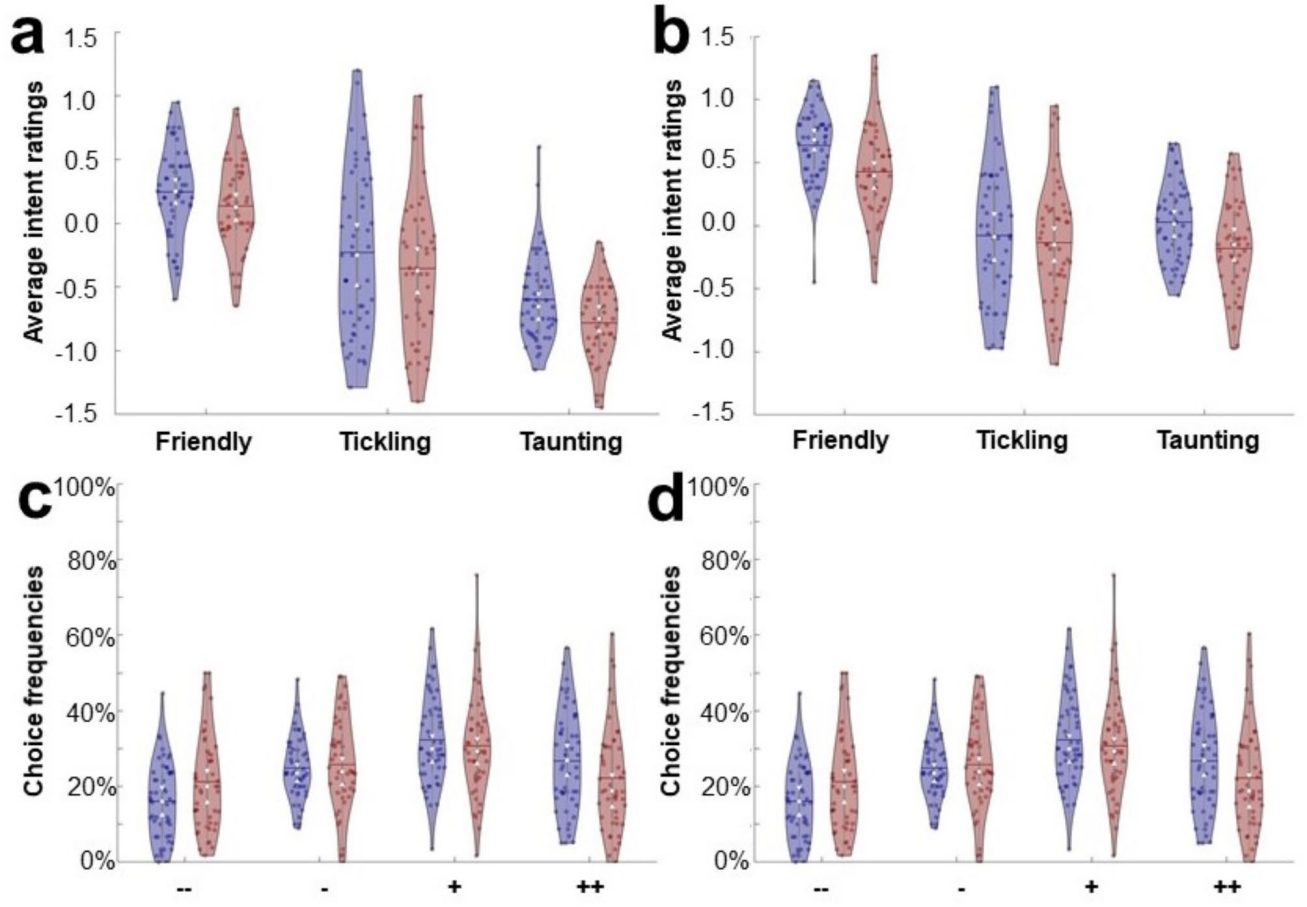
Social intent attribution. Average intent ratings of MDD patients as compared to HC during perception of auditory (a) and visual laughter (b) as well as choice frequencies for each response category (strongly positive (++), slightly positive (+), slightly negative (-), strongly negative (--)) during evaluation of auditory (c) and visual (d) laughter stimuli. Data were presented as violin plots including mean (horizontal lines), standard deviation (vertical lines), median (white circles), and 25% and 75% quartiles (white triangles) separately for MDD patients (red) and HC (blue).

Participants responded to 95.6% ± 0.8% of the trials. The 3 × 2 × 2 repeated-measures ANOVA with behavioral data (i.e. mean laughter ratings, see Fig. 1a and b) as dependent variable revealed a main effect for group (F(1,98) = 7.88, *p* < 0.01, ƞ^2^=0.07) which was driven in both sensory modalities by more negative evaluations of social intent in MDD patients (both T(98) > 2.17, both *p* < 0.01, both d > 0.42). Furthermore, a significant main effect of modality (F(1,98) = 119.40, *p* < 0.001, ƞ^2^=0.55) was found which was due to the fact that visual stimuli were on average rated more positively than auditory stimuli (T(99) = 11.06, *p* < 0.001, d = 1.02). There was also a significant main effect of laughter type (F(1,98) = 536.45, *p* < 0.001, ƞ^2^=0.85) as well as an interaction between modality and laughter type (F(1,98) = 50.86, *p* < 0.001, ƞ^2^=0.34) reflecting the fact that for auditory stimuli social intent ratings of laughter types were all significantly different (friendly > tickling > taunting, all T(99) > 6.47, all *p* < 0.001, all d > 0.28) while for visual stimuli friendly laughter was rated significantly more positive than the other two categories (both T(99) > 11.92, both d > 1.20), but no significant differences were found comparing tickling and taunting laughter (T(99) = 0.72, *p* = 0.47). No other interactions were significant.

Results of the additional exploratory analysis of choice frequencies for the four response categories are presented for HC (white bars) and MDD patients (grey bars) separately for auditory and visual stimuli (see Fig. 1c and d, respectively). We found significantly higher percentages of negative responses indicating a strongly negative (--) social intent attribution (both T(98) > 2.22, both *p* < 0.02, d = 0.50) and correspondingly lower percentages for strongly positive (++) intent attributions which scarcely failed to reach significance (both T(98) > 1.51, both *p* < 0.07). No significant differences for slightly positive (+) or slightly negative (-) social intent attributions (all T(98) < 1.32, all *p* > 0.09) were found.

**Fig. 2:**
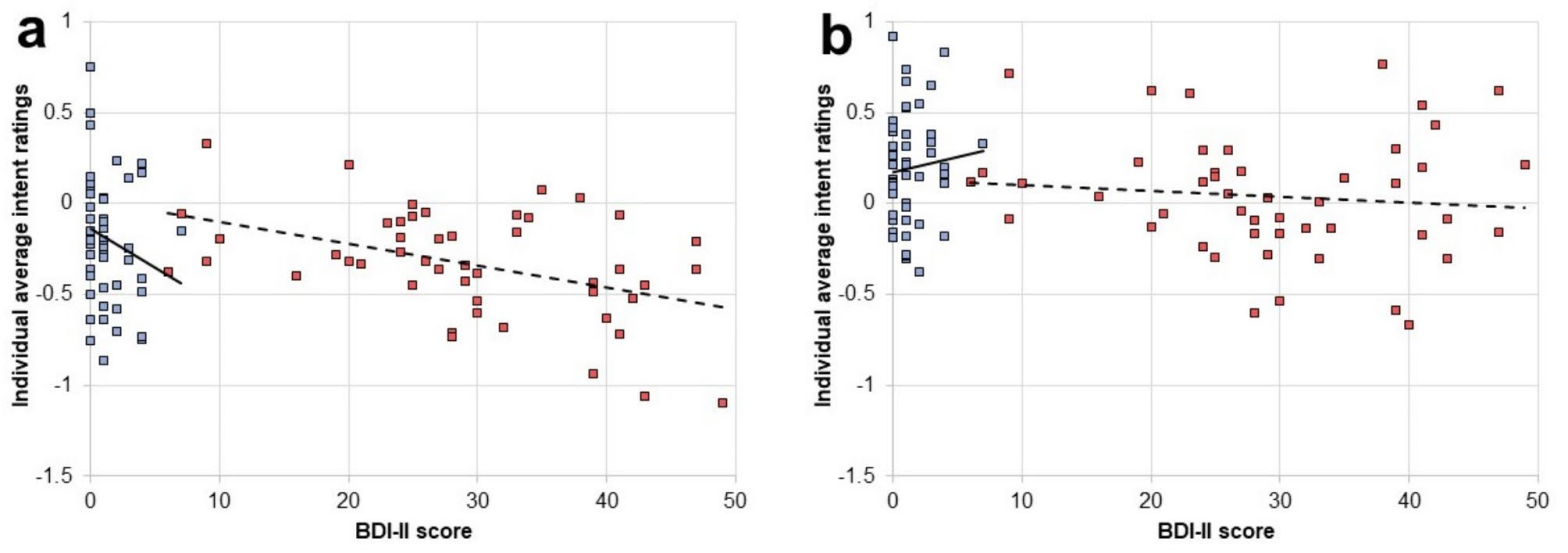
Association of attribution bias and severity of depressive symptoms. Correlation between individual average values of social intent ratings and psychometric data (i.e. BDI-II scores) during evaluation of auditory (a) and visual (b) laughter stimuli for HC (blue squares) and MDD patients (red squares). Regression lines are shown separately for HC (solid lines) and MDD patients (dashed lines).

Correlation analyses between average laughter ratings and BDI-II ratings indicated a significant linear relationship between these two variables for MDD patients (Pearson’s *r*=-0.44, *p* < 0.001) as well as HC (Spearman’s *r*=-0.24, *p* < 0.05) for auditory laughter (see Fig. 2A). No such significant linear relationship was found for visual laughter (see Fig. 2B) in neither MDD patients (Pearson’s *r*=-0.11, *p* = 0.23) nor HC (Spearman’s *r* = 0.06, *p* = 0.34). Statistical comparison revealed that the correlation with BDI-II ratings is significantly stronger for auditory than visual laughter ratings in MDD patients (z = 2.10, *p* < 0.05), but scarcely failed significance in HC (z = 1.93, *p* = 0.05). Correlation analyses between average laughter ratings of auditory stimuli and anhedonia (i.e. SHAPS) scores revealed no significant results in patients (Pearson’s *r*=-0.22, *p* = 0.06) or HC (Spearman’s *r*=-0.11, *p* = 0.21). Similarly, no significant results were obtained for the corresponding analyses using average laughter ratings of visual stimuli in MDD patients (Pearson’s *r*=-0.22, *p* = 0.07) or HC (Spearman’s *r* = 0.11, *p* = 0.22).

### Neuroimaging data

**Fig. 3:**
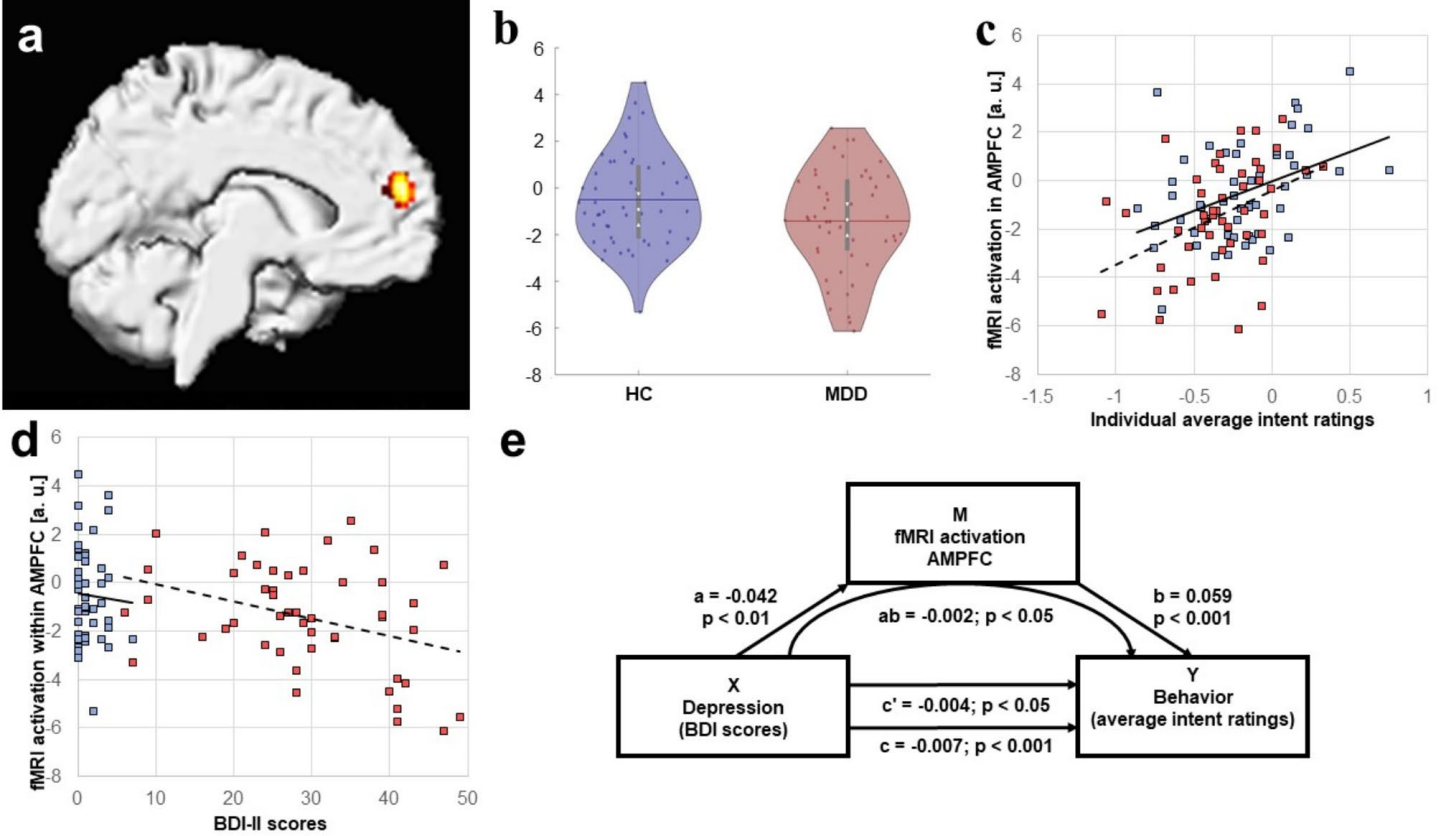
Neural correlates of the negative attribution bias in major depression. The whole-brain regression analysis between cerebral activation during perception of auditory laughter and the corresponding individual mean values of social intent ratings (a) revealed a cluster within the left AMPFC (height threshold: p < 0.001, uncorrected; cluster threshold: k > 173 voxels, p < 0.05, corrected for the whole brain). At group level, MDD patients exhibited a significantly stronger deactivation in this area as compared to healthy controls (b). Individual mean values of cerebral activation within this cluster are correlated with the mean values of social intent ratings (c) as well as negatively correlated with the respective BDI-II scores (d) for both HC (blue squares) and MDD patients (red squares). Regression lines are shown separately for HC (solid lines) and MDD patients (dashed lines). Mediation analysis (e) of the impact of depression on laughter ratings through AMPFC activation as mediator variable (M) with BDI scores as independent variable (X), and mean laughter ratings as dependent variable (Y); a = effect of X on M; b = effect of M on Y partialling out X; c = total effect of X on Y; c’ = direct effect of X on Y; ab = indirect effect of X on Y through M.

The regression analysis between brain activation during perception of auditory laughter and average ratings revealed a significant cluster within the AMPFC (MNI coordinates: x=-6, y = 56, z = 20; Z score = 4.72; cluster size = 193 voxels; *p* < 0.05, whole brain corrected) which was mostly situated within the medial part of left superior frontal gyrus (see Fig. 3a) and showed significantly stronger deactivation in MDD patients than healthy controls (T(98) = 2.20, *p* = 0.01, d = 0.43, see Fig. 3b). Averaged brain activation during rating of auditory laughter within this cluster exhibited a significant correlation (see Fig. 3c) with average laughter ratings in MDD patients (Pearson’s *r* = 0.42, *p* < 0.01) as well as HC (Pearson’s *r* = 0.42, *p* < 0.01). Furthermore, averaged activation within this cluster also yielded a significant correlation (see Fig. 3d) with BDI-II scores in MDD patients (Pearson’s *r* = 0.36, *p* = 0.01), but not HC (Spearman’s *r*= -0.25, *p* = 0.08). The mediation analysis revealed that AMPFC activation (M) partially mediated the effect of BDI-II scores (X) on laughter ratings (Y) as the indirect effect of the mediator variable was significant (ab=-0.002, *p* < 0.05, see Fig. 3E). This mediation was not complete as the direct effect of depression on behavior (c=-0.007, *p* < 0.001) remained significant after controlling for AMPF activation (c’=-0.004, *p* < 0.05).

The corresponding regression analysis between activation during perception of visual laughter and average ratings did not reveal any significant cluster. At an uncorrected height threshold of *p* < 0.001 an activation cluster within the medial part of the right frontal lobe was found (MNI coordinates: x = 8, y = 28, z = 2; Z score = 4.36; cluster size = 74 voxels; *p* = 0.38, whole brain corrected). However, this cluster was mostly located within the white matter (WM) of the corpus callosum and only a minority of voxels extended into the adjacent medial prefrontal cortex belonging to the subgenual anterior cingulate cortex (ACC) according to AAL.

## Discussion

We investigated whether laughter is evaluated differently by MDD patients versus HC regarding the expressed social intent and if so whether it is possible to define a link for this behavioral result to depression severity and brain activation. MDD patients rated laughter significantly more negative than HC and this effect occurred similarly for visual and auditory laughter. This shift in rating of laughter observed in MDD is in line with previous studies on other types of emotional signals including facial expressions^18,19^ and prosody^20,29^. Moreover, meta-analyses identified depression severity as measured by BDI scores as moderator variable for this behavioral effect^19,20^ which is in line with our results which revealed a stronger relationship for general depression severity than anhedonia which can also impact neural activation^30^. Surprisingly, however, ratings of social intent in our study correlated with depression severity only for auditory, but not visual laughter. A possible explanation for this difference across modalities is the strong positivity bias in purely visual stimuli. While presenting exclusively the sound clips or the mute visual video clips of the very same stimulus material is certainly a technical sound method to present separately the visual or the auditory component of the stimuli, this has different effects on the perceived social intent expressed by laughter. While presenting the auditory sound clip alone or in combination with the visual component results in nearly identical evaluations of perceived social intent (see methods reporting the prestudy on possible effects of communication channel to evaluate the stimuli employed during fMRI), omission of the auditory information by presenting mute video clips leads to significantly more positive ratings in both MDD patients and HC. The results of the prestudy indicate that evaluation of social intent expressed by laughter is dominated by the auditory component and that its omission clearly impedes differentiation of benign versus malign laughter. This finding concurs with a previous report on evaluation of laughter regarding authenticity which similarly showed that auditory affective cues have a greater influence on multimodal percepts than visual cues^13^. It should be noted, however, that this auditory dominance in evaluation of laughter does not occur in all aspects of laughter evaluation, as e.g. discrimination of laughter from crying has been found to be more effective based on visual than on auditory information^31^. The more positive social intent attributions for visual stimuli found in our study does not infer that the employed visual stimuli are ecologically invalid as there are situations during which laughter can only be perceived visually (e.g. during observation through a sound proof window), but that humans are simply not well equipped to correctly decode information regarding social intent expressed by laughter in purely visual stimuli (possibly as situations with purely visual signialling are rather rare and thus of limited survival value).

Analysis of fMRI data revealed a linear relationship between rating of social intent of auditory laughter stimuli and activation within the AMPFC which is in line with a previous fMRI study identifying this area for processing emotions in laughter expressed in the auditory domain^10^. Within the neuroscientific literature different nomenclatures exist to characterize functional subdivisions of the medial prefrontal cortex, but a common denominator is that it consist of three parts including dorsomedial (or posterior rostral), anteromedial (or anterior rostral) and ventromedial (or orbital) regions which functionally extend also into the neighboring ACC (for a review, see^15,32^). The AMPFC is thought to subserve self-knowledge, person knowledge, and mentalizing^15^ or self-related and affective processes^32^ including emotion regulation^16^. Concerning affective processing AMPFC has been demonstrated to contain supramodal representations of emotional information conveyed via voice, face, or body posture^33^. Surprisingly, however, the linear relationship between behavior and fMRI activation in AMPFC was restricted to the auditory domain. The corresponding analysis for visual laughter stimuli revealed a cluster within the subgenual ACC which has been previously reported to induce bursts of laughter following electical stimulation^34^ as well as adjacent corpus callosum. As noted above, functional partitions of the medial prefrontal cortex do include neighboring ACC structures as activations in social cognition tasks are often found on both sides of the cingulate sulcus (e.g., see Fig. 3 in^15^). We still report the cluster found during processing of visual laughter only descriptively (for usage in future meta-analyses) for two reasons: First, this cluster failed to reach significance after correction for multiple comparisons across the brain, and second, it was mostly located within the WM of the corpus callosum. It should be noted, however, that a meta-analysis on processing emotionally valenced facial stimuli revealed activation differences between MDD patients and HC in exactly this location (compare Fig. 3 in^35^). Furthermore, recent findings^36^ question the traditional view that changes of fMRI signals within the WM reflect meaningless noise (which was consequently regressed out as nuisance covariate in many previous fMRI studies), but indicate that WM structures can similarly show activation changes which occur time-locked to the experimental design (i.e. stimulus presentation or task demands). Therefore, it is possible that rating of social intent expressed by auditory and visual laughter are represented in spatially distinct areas within AMPFC and subgenual ACC, respectively. An alternative interpretation, however, is that the general shift towards more positive social intent induced by purely visual laughter is responsible for this separate location of activity. A possible way to examine this issue in future studies could be to include an audiovisual condition in the experimental design and test whether spatially distinct representations are restricted to visual stimuli.

Several limitations have to be considered regarding interpretation of the results of our study: First, the MDD patients were recruited from spezialized wards for treatment of MDD and the majority received antidepressants. It would be interesting to evaluate potential effects of medication on laughter perception in future studies. Second, as we examined our patients only once, we cannot make inference on whether a more negative evaluation of laughter disappears or persists after successful treatment. Third, the stimuli was created in cooperation with professional actors which has the advantage that experimental factors can be well controlled (e.g. recognizability of the stimuli across categories) and it would be difficult to create spontaneous taunting laughter for subsequent experimental use. However, it should be noticed that authenticity can modulate brain activity during laughter perception^37^. Finally, we focused on laughter perception and particularly on the AMPFC as potential module for processing socially relevant information in laughter, but it should be kept in mind that the neural ciruits underlying the neurobiology of laughter include widespread networks (e.g. frontolimbic and motor regions for laughter production^38,39^).

In summary, this is the first study examining evaluation of laughter stimuli as well as their neural representation in MDD patients and HC. In both sensory modalities, social intent was rated more negatively by MDD patients. A significant correlation between rating and depression severity was only found for auditory, but not visual laughter. Activation within the AMPFC was reduced in MDD patients, correlated both with behavior as well as BDI-II scores and mediation analyses provided evidence that more negative perception of laughter in depression is at least partially mediated via this neural structure. Future studies should evaluate whether this effect is restricted to laughter or can be similarly observed for evaluation of other types of auditory stimuli (e.g. eating noise signaling either approval or rejection of served food) as well as potential influences and interactions regarding gender, personality, and cultural background of the persons expressing and perceiving laughter. Finally, laughter therapy has been proposed for treatment of MDD (for a review, see^40^), but clinicians as well as patients should be informed that MDD can result in a tendency towards a more negative interpretation of laughter which might help to avoid misunderstandigs during conversation as well as treatment. In conclusion, these findings on social intent attribution during laughter perception provide further evidence for a negative interpretation bias of self-referential social cues in patients with MDD. Furthermore, the observed activation pattern within the AMPFC advances the understanding of the neurobiological processes mediating this cognitive bias.

## Methods

The experiment has already been described for healthy participants^14^. The current study additionally includes MDD patients, but is otherwise analogous to the previous study in healthy participants.

### Participants

48 MDD patients and 52 HC were included in the study. All participants were right-handed as determined by the Edinburgh Handedness Inventory^41^, native speakers of German, and were screened by trained psychologists to exclude comorbid psychosis, current substance abuse, and personality disorders as well as past or current neurological diseases. MDD patients were recruited via the specialized psychotherapy wards of the University Clinic for Psychiatry Tübingen and healthy participants were recruited via public announcements. MDD patients fulfilled the criteria for recurrent MDD with a current episode duration of at least two weeks. Most MDD patients (42/48) received antidepressive medication.

Demographic data (gender, age, partnership status and intake of psychotropic medication) as well as psychometric data including verbal intelligence as assessed by the Multiple Choice Vocabulary Intelligence Test (MWT-B)^42^, self-rating of depression symptoms using Beck Depression Inventory (BDI-II)^43^, external-rating of depression symptoms using the Hamilton Depression Rating Scale (HRSD)^44^, and self-rating of anhedonia using the Snaith-Hamilton-Pleasure-Scale (SHAPS)^45^ are presented in Table 1. Interval scaled data are reported as mean ± standard error of the mean (SEM).

Gender, parternship status, and psychotropic medication were compared using the chi-squared test. Age and MWT-B-scores were compared using t-tests for independent samples. Psychometric differences assessed by BDI-II, HRDS, and SHAPS were statistically tested using the Mann-Whitney-U-test as the Shapiro-Wilk test indicated that data significantly deviated from normal distribution in HC (all *p* < 0.001), but not MDD patients (all *p* > 0.18). The study conforms to the Code of Ethics of the World Medical Association (Declaration of Helsinki). It was approved by the Ethics Committee of the Medical Faculty of the University of Tübingen. All participants gave written informed consent to the study prior to participating.

### Stimulus material

The stimulus material was generated in cooperation with eight professional actors portraying three laughter types (friendly laughter, tickling laughter, and taunting laughter) to obtain audiovisual (AV) movie clips (duration 1.5 s) from which auditory (A) sounds and mute visual (V) movies were obtained (i.e. both types of stimuli were created from the same stimulus set). The actors wore black head caps against a black background to minimize the influence of visual cues that were not part of the human face. The video sequences were post-processed using Adobe Premiere Pro CS3 software to guarantee the alignment of the vertical facial symmetry axis and similar size of the portrayed faces. Mean sound intensity was set to 70 dB using PRAAT, version 5.1.07^46^. The obtained stimulus set consisted of 187 stimuli (for a detailed description of the stimulus material, see^47–49^) and was evaluated in two behavioral prestudies by healthy participants with regard to the recognizability of the expressed laughter type (*n* = 14, mean age 24.4 ± 2.4 years) and authenticity (*n* = 14, mean age 24.3 ± 3.5 years). Only stimuli with recognition rates exceeding chance level and with at least average authenticity ratings (i.e., > 3.0 on a 9-point-scale; mean authenticity 5.1 ± 1.4) when presented audiovisually were selected for the fMRI study (20 stimuli for each of the three laughter types) which were balanced for the gender of the actors (11 and 9 stimuli of female.

and male actors for each laughter type, respectively). In another prestudy, the social intention of these 60 stimuli was evaluated by 18 healthy subjects (7 females, mean age: 25.8 ± 3.3 years) to examine possible effects of the communication channel (A, V, AV) on the perceived social intention. Participants were asked to rate the social intention of the presented laughter on a four-point scale as strongly positive (++), slightly positive (+), slightly negative (-), or strongly negative (--). Mean values of laughter rating were calculated by multiplying the respective number of responses of the four response categories with + 1.5 (++), + 0.5 (+), -0.5 (-), and − 1.5 (--) and dividing the obtained sum of the four categories by the number of total responses (i.e. excluding trials with missed responses) for A, V, AV stimuli separately. Visual stimuli were rated more positive than auditory and audiovisual stimuli (V stimuli: 0.17 ± 0.06, A stimuli: -0.12 ± 0.05, AV stimuli: -0.12 ± 0.06) which was significant (both T(17) > 3.58, both *p* < 0.01) while no significant difference was found by comparing auditory and audiovisual stimuli (T(17) = 0.13, *p* = 0.90).

### Experimental design

During fMRI we employed only A and V stimuli, but not the AV stimuli from which both classes of stimuli were generated to avoid crossmodal effects that might be induced by presentation of bimodal stimuli preceding unimodal stimuli as well as order effects that would result if AV stimuli were always presented following the unimodal stimuli. 30 A and 30 V stimuli were presented in each of two sessions in fully randomized order (i.e. each stimulus was presented twice) with an inter-trial interval ranging from 9 to 12 s (jittered in steps of TR/4). Moreover, each session included six randomly inserted null events. We did not inform the study participants that the A and V stimuli were created from the same AV stimulus set. Furthermore, we emphasized that there were no “right” or “wrong” responses, but that the study aim is to assess their individual perception of the social intent expressed by laughter. The scale was flipped in horizontal direction for half of the study participants to avoid lateralization effects due to motor responses. Visual stimuli were presented via an MRI compatible monitor (Optostim, Medres Medical Research, Köln, Germany) positioned approximately two meter behind the participants head and a mirror system mounted to the head coil. Auditory stimuli were presented via MRI compatible headphones (MR Confon GmbH, Magdeburg, Germany). Stimulus presentation and recording of behavioral responses relied on Presentation software (Neurobehavioral Systems, http://www.neurobs.com/). Participants conveyed their decision regarding social intent using a four-finger MRI-compatible response system (Celeritas Fiber Optic Button Response System, Psychology Software Tools).

### Analysis of behavioral data

Only responses occurring within 5s of stimulus onset were included in the analysis. Mean values of laughter rating were calculated for HC and MDD patients as well as for visual and auditory laughter stimuli separately. The Shapiro-Wilk-test indicated no evidence for non-normal distributions (all *p* > 0.34). Thus, we submitted the mean rating values to a repeated-measures analysis of variance (ANOVA) with modality (visual, auditory) and laughter type (friendly, tickling, taunting) as within-subjects factor and group (HC, MDD patients) as between-subjects factor. Significant results in the ANOVA were further examined by posthoc t-tests regarding differences between groups, modality, and laughter categories. We additionally calculated the mean percentage of responses for the four response categories separately for HC and MDD patients and visual and auditory laughter stimuli. These mean percentages of responses were exploratively compared between groups using two-sided t-tests for independent groups with unequal distribution. The Shapiro-Wilk test was used to test for significant deviations from non-normal distributions. Correlation analyses between laughter rating and BDI scores as well as SHAPS scores were carried out using Pearson’s correlation if the Shapiro-Wilk test did not show significant deviations from non-normal distributions for any of the included variables. If at least one variable was non-normally distributed, Spearman’s correlation analyses were employed. Comparison of correlation coefficients was based on Meng, Rosenthal, and Rubin’s z^50^. Computation of effect sizes relied on Cohen’s d and ф-values for comparison of interval scaled and categorical variables, respectively, and parial ƞ^2^-values for ANOVAs.

### Image data acquisition

We acquired structural T1-weighted images (TR = 2.3 s, TE = 4.16 ms, TI = 0.9 s, voxel size: 1 × 1 × 1 mm^3^) and functional images (478 images per session, 72 slices, slice thickness 2 mm + 1 mm gap, TR = 1.5 s, TE = 34 ms, voxel size: 2 × 2 × 2 mm^3^, multi-band acceleration factor 3) at a 3T scanner (PRISMA, Siemens, Erlangen, Germany) equipped with a 20 channel head coil. In addition, a field map (36 slices, slice thickness 3 mm + 1 mm gap, TR = 0.4 s, TE(1) = 5.19 ms, TE(2) = 7.65 ms, voxel size: 3 × 3 × 3 mm^3^) was obtained to correct for possible image distortions.

### Analysis of imaging data

Statistical parametric mapping software (SPM12, Wellcome Trust Center for Neuroimaging, UCL, London, UK, https://www.fil.ion.ucl.ac.uk/spm/software/) was used for imaging data analysis. Preprocessing included slice time correction, realignment, unwarping to correct for field distortions and to remove residual movement-related variance due to interactions between motion and field distortions^51^, normalization to MNI space (Montreal Neurological Institute, resampled voxel size: 2 × 2 × 2 mm^3^) based on the unified segmentation in SPM^52^ and smoothing with a Gaussian filter (6 mm full width at half maximum). The first five EPI images of each session were discarded from analysis to exclude measurements preceding T1 equilibrium. Statistical analysis relied on a general linear model with separate regressors for each of the 120 trials using a stick-function time locked to the onset of stimulus presentation and convolved with the hemodynamic response function. To remove low-frequency components, a high-pass filter with a cutoff frequency of 1/128 Hz was used. Serial autocorrelations were accounted for by modeling residual errors as a first-order autoregressive process with a coefficient of 0.2^53^ plus a white noise component^54^. Individual contrast images representing main effects were calculated by adding activation for each trial (i.e. beta images) during which participants responded (i.e., trials with missed responses were excluded from the analysis) and divided by the number of included trials. These contrast images generated at first level were submitted to second-level simple regression analyses using subject-specific mean values of laughter rating separately for visual and auditory trials. Additional contrasts regarding modality effects and differences between laughter types are reported in the supplemental data. Assignment of anatomical structures relied on the automatic anatomical labeling tool integrated in SPM^55^. All activations are reported using an uncorrected height threshold of *p* < 0.001. Correction for multiple comparisons was carried out at cluster level using an extent threshold of k ≥ 178 voxels (*p* < 0.05, family-wise error (FWE) corrected). Within significant clusters we compared the activation between groups (HC versus MDD patients) and calculated correlation coefficients between brain activations and average laughter ratings as well as brain activation and psychometric data (i.e. BDI-II scores and SHAPS scores) separately for the two groups of MDD patients and HC. Finally, it was examined in a mediation analysis whether activation of significant clusters serves as mediator variable (M) on the association between the independent variable (X) depression (i.e. BDI-II scores) and the dependent variable (Y) behavioral ratings using a bootstrapping approach with 5000 resamples based on the SPSS implementation PROCESS 4.2 (model 4). PROCESS is a regression-based analysis tool for mediation, moderation and conditional process analyses. We estimated an indirect effect in a simple mediation model^56^.

**Table 1:** Demographic and psychometric data.

	MDD patients	healthy controls	*p* value / effect size
Gender (female/male)	25/23	27/25	n.s.
Age in years (mean ± SEM)	33.2 ± 1.7	29.2 ± 1.3	n.s.
Partnership status (single/in partnership)	32/17	18/33	< 0.01 / ϕ = 0.30
Antidepressive medication (yes/no)	42/6	0/52	< 0.001 / ϕ = 0.88
MWT-B scores (mean ± SEM)	107.5 ± 2.0	106.3 ± 2.1	n.s.
BDI-II scores (mean ± SEM)	29.2 ± 1.6	1.3 ± 0.2	*p* < 0.001 / d = 1.75
HRSD scores (mean ± SEM)	21.7 ± 0.8	0.5 ± 0.1	*p* < 0.001 / d = 1.87
SHAPS scores (mean ± SEM)	18.6 ± 0.9	3.4 ± 0.5	*p* < 0.001 / d = 1.64

MDD: Major depressive disorder SEM: standard error of the mean; MWT-B: Multiple Choice Vocabulary Intelligence Test; BDI-II: Beck Depression Inventory; HRSD: Hamilton Depression Rating Scale; SHAPS: Snaith-Hamilton-Pleasure-Scale.

## Electronic supplementary material

Below is the link to the electronic supplementary material.

Supplementary Material 1

## Data Availability

All neuroimaging and behavioral data acquired in this study are available on reasonable scientific request from the corresponding autor (Email: Thomas.Ethofer@med.uni-tuebingen.de).
