# Supplementary material for: Neural processing of laughter in depression
Source: Sci Rep. 2025 Apr 27;15:14724. doi: 10.1038/s41598-025-97385-6 (PMC12034789; doi:10.1038/s41598-025-97385-6)
Supplement: Supplementary file 1 — Supplementary Material 1 [file 41598_2025_97385_MOESM1_ESM.pdf]

Supplemental Data

All imaging analyses reported in the supplemental data were corrected for multiple comparisons across the whole brain ($p<0.05$, corrected at extent threshold with a cluster size, $k>120$ voxels). To further characterize the current imaging data set we calculated the main effects versus baseline for visual and auditory stimulation (V+A), visual stimulation (V), and auditory stimulation (A) and as well as the contrasts V-A and A-V. As expected, the main effects versus baseline revealed a broad network of activation in sensory, motor, and higher order cognitive brain areas. However, due to merging of activation clusters no meaningful labeling of activation clusters was possible for the main effects (see Supplemental Figure 1A-C and Supplemental Table 1). The sensory specific contrasts yielded in bilateral visual and auditory cortex, respectively (see Supplemental Figure 1D-E and Supplemental Table 1). Interestingly, the contrast A>V showed also activation in the AMPFC which presumably indicates a stronger involvement of this structure during rating of social intent of auditory as compared to visual laughter. Smaller activation clusters were labeled using the two brain regions with the largest amounts of activated voxels (as determined by AAL). Neither main effects nor contrasts yielded significant differences between HC and MDD patients in whole brain comparisons.

Moreover, we also calculated laughter type specific contrasts separately for visual and auditory laughter by comparing the two emotional laughter types (EMO, i.e. friendly and taunting laughter) with tickling laughter (TIC, see Supplemental Figure 2A-D and Supplemental Table 2). Comparison of visual TIC > visual EMO revealed a widespread network including bilateral motion-sensitive cortices in posterior middle temporal cortex (i.e. area V5, for a review see 1) as well as the bilateral cortex adjacent to the posterior superior temporal sulcus which represents a central hub for biological motion processing (for a review, see 2). Furthermore, the contrast visual TIC > visual EMO yielded smaller activation clusters in bilateral inferior frontal/insular cortices which are part of the salience network (for a review, see 3) and the occipital poles in primary visual cortices. The reverse contrast visual EMO > TIC resulted in significantly stronger activation in right angular/inferior parietal cortex which has been previously identified to contain representations of emotions in facial expressions (4). The contrast auditory TIC > auditory EMO showed significantly stronger activations along bilateral superior/middle temporal cortices which has been previously described for comparison of these laughter types in the auditory domain (5). The reverse contrast auditory EMO versus auditory TIC did not reveal any significant suprathreshold clusters which contrasts with previous results on processing auditory laughter stimuli (5) yielding activations in the AMPFC for this comparison. The most probable explanation for this difference across studies is the employed task of explicit

rating of social intent expressed by laughter in the present study. In the study of Szameitat and colleagues (5) participants were instructed to identify the laughter type or count the number of laughter bouts. Thus, an implicit rating of social intent of friendly and taunting laughter, but not tickling laughter might have resulted in an AMPFC activation in the previous study (5) which did not occur in the present study as rating of social intent was explicitly required after each stimulus (which might even increase processing demands during evaluation of subtle variations of social intent in tickling laughter as compared to the other two laughter types). None of the four contrasts of laughter types yielded significant differences between HC and MDD patients in whole brain comparisons.

Supplemental References

1. Zeki S (2015): Area V5-a microcosm of the visual brain. *Front Integr Neurosci*. 9:21.
2. Bachmann J, Munzert J, Kruger B (2018): Neural Underpinnings of the Perception of Emotional States Derived From Biological Human Motion: A Review of Neuroimaging Research. *Front Psychol*. 9:1763.
3. Uddin LQ (2015): Salience processing and insular cortical function and dysfunction. *Nat Rev Neurosci*. 16:55-61.
4. Kim J, Schultz J, Rohe T, Wallraven C, Lee SW, Bulthoff HH (2015): Abstract representations of associated emotions in the human brain. *J Neurosci*. 35:5655-5663.
5. Szameitat DP, Kreifelts B, Alter K, Szameitat AJ, Sterr A, Grodd W, et al. (2010): It is not always tickling: distinct cerebral responses during perception of different laughter types. *Neuroimage*. 53:1264-1271.

Supplemental Tables

Supplemental Table 1: Main effects and contrasts between sensory modalities

Anatomical region	MNI coordinates	T value	Z score	cluster size
Main Effect A+V:				
N/A*	36 22 -2	23.80	N/A**	88588
Main Effect V:				
N/A*	40 -62 -18	25.08	N/A**	80938
Main Effect A:				
N/A*	-38 -28 10	25.21	N/A**	84463
Contrast V>A:				
Bilateral visual cortex	-40 -76 -14	23.17	N/A**	27631
Right inferior/middle frontal cortex	46 8 28	12.53	N/A**	2280
Bilateral anterior/middle frontal cortex	4 2 8	8.31	7.22	147
Contrast A>V:				
Bilateral auditory cortex	42-24 10	23.32	N/A**	32754
Bilateral cerebellar cortex	28 -60 -26	11.43	N/A**	5003
Left postcentral/precentral cortex	-48 -14 44	9.17	7.28	683
Right precentral/postcentral cortex	24 -28 66	6.99	6.28	269
Left postcentral/paracentral cortex	-18 -32 58	6.20	5.69	258
Left precentral/paracentral cortex	-20 -16 64	5.65	5.24	253

*No meaningful anatomical labeling possible due to merging of several activation clusters

**SPM does not provide valid Z scores for T values >10

Supplemental Table 2: Contrasts between laughter types

Anatomical region	MNI coordinates	T value	Z score	cluster size
Contrast: TIC > EMO (Visual)				
Right middle/inferior temporal cortex	46 -60 4	7.30	6.51	711
Left middle/superior temporal cortex	-42 -74 2	7.26	6.49	1542
Right superior/middle temporal cortex	50 -36 6	6.51	5.92	1085
Left inferior frontal/insular cortex	-34 26 2	5.76	5.34	585
Left middle/superior occipital cortex	-12 -96 10	5.04	4.75	199
Right insular/inferior frontal cortex	32 30 0	4.94	4.66	133
Right superior/middle occipital cortex	18 -96 14	4.32	4.13	124
Contrast: EMO > TIC (Visual)				
Right angular/inferior parietal cortex	48 -62 46	4.66	4.42	387
Contrast: TIC > EMO (Auditory)				
Left superior/middle temporal cortex	-48 -14 2	9.08	7.72	2678
Right superior/middle temporal cortex	52 -2 -6	8.48	7.34	3066
Left inferior frontal/insular cortex	-34 30 2	4.73	4.48	150
Contrast: EMO > TIC (Auditory)				
No suprathreshold clusters				

Supplemental Figures:

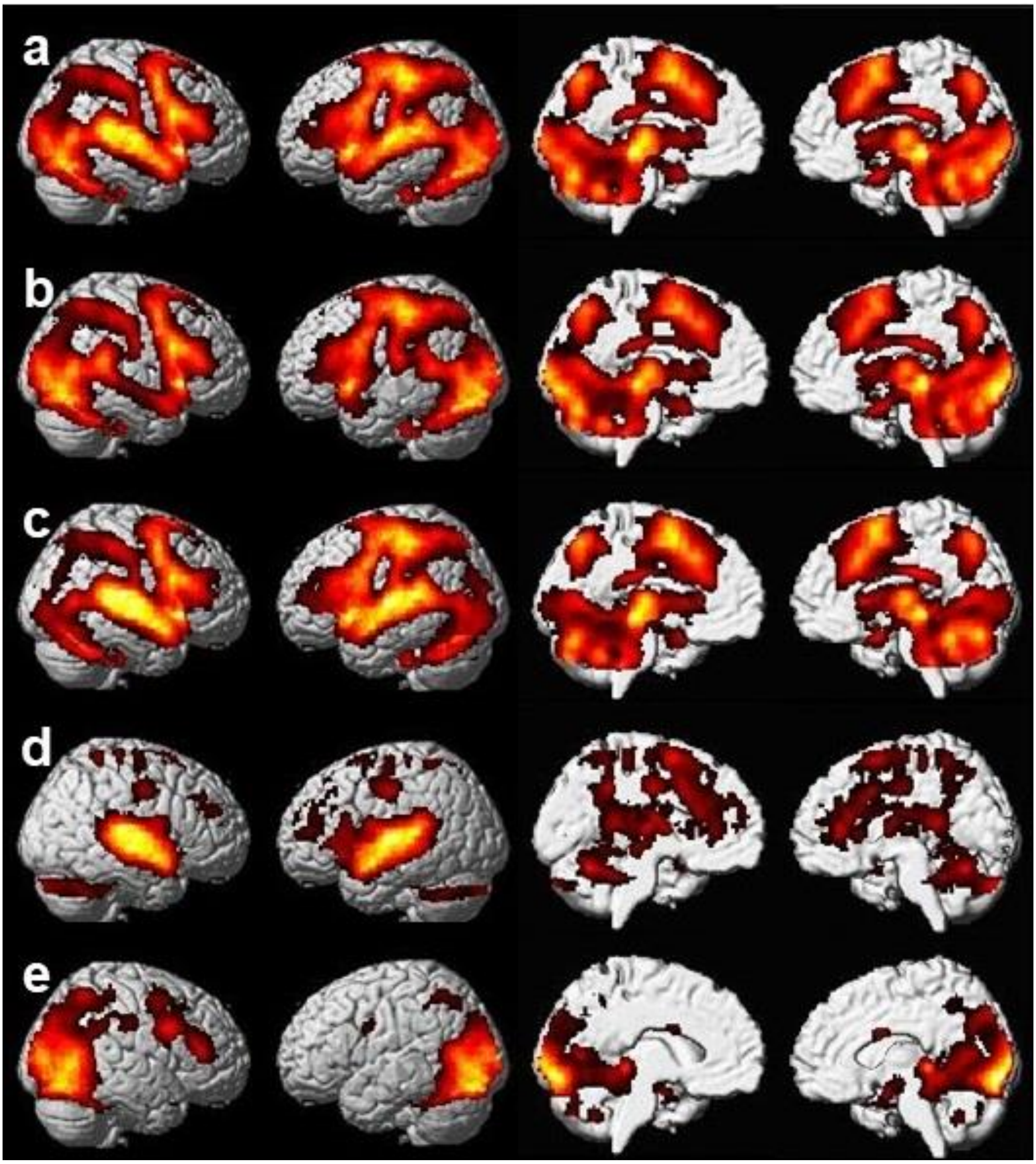

Supplemental Figure 1: Neural activation dependent on sensory modality. Main effects of auditory and visual stimulation (a), visual stimulation (b), auditory stimulation (c), differential contrasts of visual stimulation > auditory stimulation (d) and differential contrast of auditory stimulation > visual stimulation (e). All activations are reported on a significance threshold of $p<0.05$ (corrected at extent threshold $k>120$ voxels).

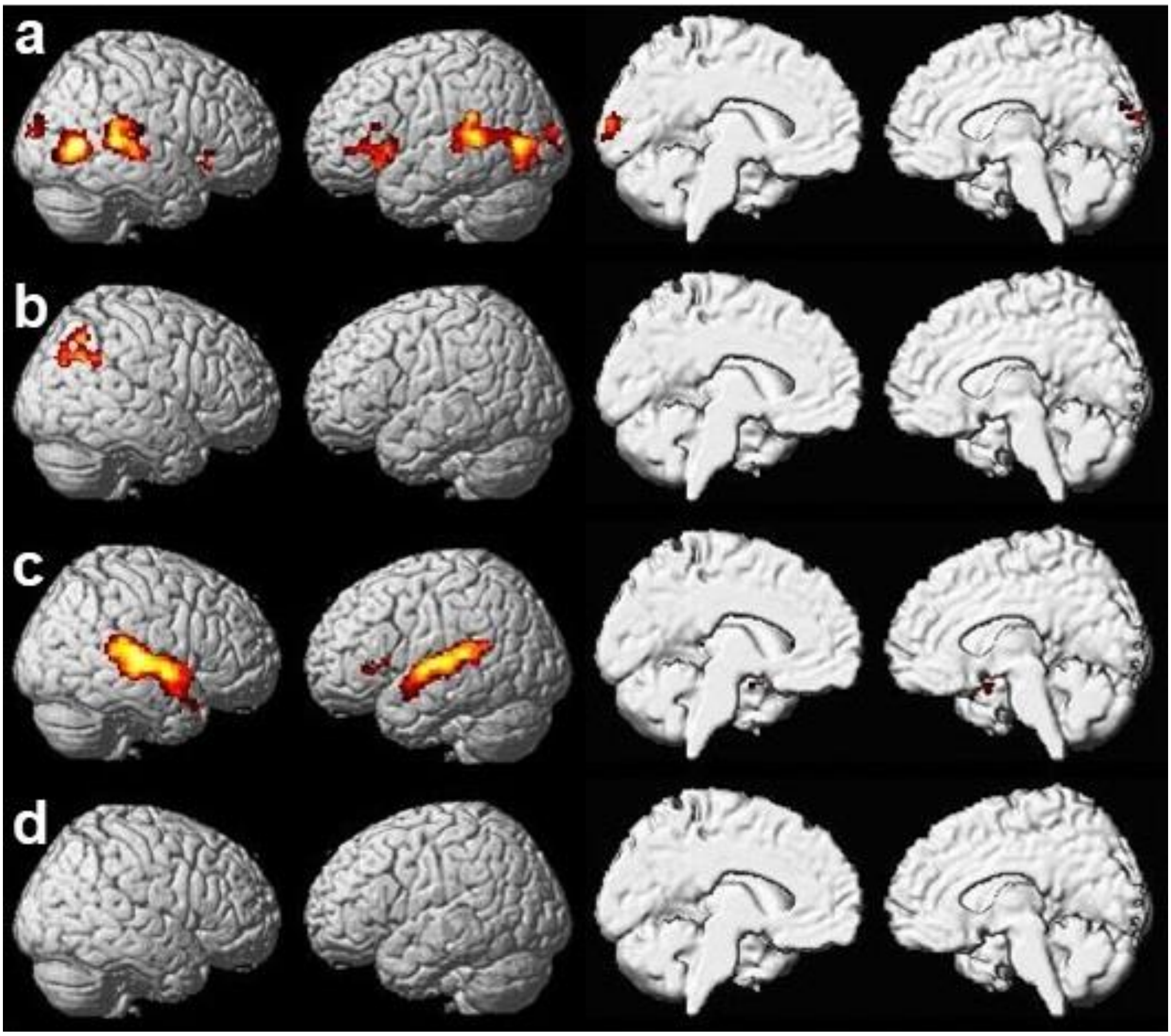

Supplemental Figure 2: Neural activation dependent on laughter type. Tickling laughter (TIC) was contrasted with emotional laughter (EMO, i.e. friendly and taunting laughter). Contrasts of visual TIC > visual EMO (a), visual EMO > visual TIC (b), auditory TIC > auditory EMO (c), and auditory EMO > auditory TIC (d). All activations are reported on a significance threshold of $p<0.05$ (corrected at extent threshold $k>120$ voxels).
